# Increased frequency of PD-1^hi^CXCR5^-^ T cells and B cells in patients with newly diagnosed IgA nephropathy

**DOI:** 10.1038/s41598-019-57324-8

**Published:** 2020-01-16

**Authors:** Xin Wang, Tao Li, Rui Si, Jinyun Chen, Zhihui Qu, Yanfang Jiang

**Affiliations:** 1 Key Laboratory of Organ Regeneration & Transplantation of the Ministry of Education, Genetic Diagnosis Center, The First Hospital of Jilin University, Changchun, 130021 China; 2 Key Laboratory of Zoonoses Research, Ministry of Education, The First Hospital of Jilin University, Changchun, 130021 China; 3 Department of Nephrology, Chinese PLA General Hospital, Chinese PLA Institute of Nephrology, State Key Laboratory of Kidney Diseases, National Clinical Research Center of Kidney Diseases, Beijing Key Laboratory of Kidney Disease, Beijing, China; 4 Yanbian University medical College, Yanbian, 133002 China; 5 Department of Nephrology, The First Hospital of Jilin University, Changchun, 130021 China

**Keywords:** Autoimmunity, Minimal change disease

## Abstract

Recent research has identified a population of PD-1^hi^CXCR5^−^ ‘peripheral helper’ T (Tph) cells that simulate plasma cell differentiation by interactions between IL-21 and SLAMF5. However, the alteration of circulating Tph and CD138^+^ B in IgA nephropathy (IgAN) remains poorly understood. Flow cytometry analysis was used to measure the frequency of circulating PD-1^hi^CXCR5^−^ T cells and CD138^+^ B cells in 37 patients with IgAN and 23 healthy controls (HCs). Estimated glomerular filtration rate (eGFR), 24 h urinary protein and serum cytokine concentrations were measured. The percentage of different subsets of circulating PD-1^hi^CXCR5^−^ T cells and CD138^+^ B cells were significantly higher in patients with IgAN compared to HCs. Pretreatment, the percentage of different subsets of circulating PD-1^hi^CXCR5^−^ T cells and CD138^+^ B cells were negatively correlated with eGFR, the percentage of circulating CD138^+^ B cells was positively correlated with 24-h urinary protein concentration, and the percentage of circulating PD-1^hi^CXCR5^−^, CD28^+^ and ICOS^+^ T cells. Posttreatment, the percentage of different subsets of circulating PD-1^hi^CXCR5^−^ T cells and CD138^+^ B cells and serum IL-21 concentration were significantly reduced. Different subsets of circulating PD-1^hi^CXCR5^−^ T cells contribute to the progression and pathogenesis of IgAN by regulating the differentiation of CD138^+^ B cells through a combination of surface molecules.

## Introduction

IgA nephropathy (IgAN) is the most common form of glomerulonephritis (GN), affecting up to 30–45% of all patients with primary GN in Asia^1,2^. IgAN is a chronic progressive disease; an estimated 25–30% of patients require renal replacement therapy within 20–25 years of initial IgAN diagnosis, while 1.5% of patients with IgAN reach end-stage kidney disease (ESKD) each year^3^.

IgAN is characterized by deposition of IgA and IgG antibodies in the mesangium and the presence of immune complement complexes in the glomeruli^2^. Currently, the mechanisms underlying the pathogenesis of IgAN are unknown, but studies to-date show it to be a mutlifactorial process^4,5^.

T cells promote IgA production^6^ and mediate the course of IgAN disease. However, the roles of the different CD4^+^ helper T cell types in the pathogenesis of IgAN remain to be elucidated. Some evidence suggests that Th2 cytokines control the abnormal glycosylation of IgA1 in IgAN^7^, and other studies show a Th1 predominance^8^.

Recent research has identified a population of PD-1^hi^CXCR5^−^ ‘peripheral helper’ T (Tph) cells that are distinct from PD-1^hi^CXCR5^+^ T follicular helper (Tfh) cells. Tph cells express Blimp1 and the inflammatory chemokine receptors CCR2, CCR5, and CX3CR1 and simulate plasma cell differentiation by interactions between IL-21 and SLAMF5^9,10^. Tph cells support B-cell responses and antibody production in pathologically inflamed non-lymphoid tissues^9^. A previous study reported a high frequency of CXCR5^+^CD4^+^ Tfh cells in patients with minimal change disease^11^. However, information about the frequency of PD-1^hi^CXCR5^−^ T cells and B cells in patients with IgAN is scarce.

In this study, we analyzed the percentage of various subsets of circulating PD-1^hi^CXCR5^−^ T cells and CD138^+^ B cells in adults with IgAN, and investigated their potential associations with clinical parameters before and after treatment to explore the putative contribution of Tph and CD138^+^ B cells to IgAN.

## Materials and Methods

### Study subjects

Patients with IgAN identified at our institution between November 2017 and July 2018 were eligible for this study. Inclusion criteria were (1) estimated glomerular filtration rate (eGFR) <60 mL/min/1.73 m^2^ calculated using the revised eGFR formula^12^ or ratio of urinary albumin/creatinine >30 for more than 3 months; and (2) IgA-related nephrologic damage in biopsied kidney tissues, according to Lee’s grading system^13^. Exclusion criteria were complications (progressive IgAN, secondary IgAN, such as Henoch–Schonlein purpura, lupus nephritis, and other primary GN, pregnant or planning a pregnancy, diabetes mellitus, neoplasia, active peptic ulcer disease, viral hepatitis, or recent infection), selected according to published reports^14^. Age- and gender-matched healthy controls (HCs) with no history of chronic illness or recent infection were recruited from the Physical Examination Center of the same hospital.

All study subjects provided written informed consent. The experiment was approved by The Ethics Committee of First Hospital of Jilin University (Registration Number: 2017-370, Date of Registration: October 11, 2017). All methods were performed in accordance with the relevant guidelines and regulations.

### Treatment and follow up

Treatment for patients with IgAN and 24-h urinary protein >1 g included pednisone (PDN, Tianyao Pharmaceuticals, Tianjin, China) 1 mg/kg/d for the first two months, gradually decreasing to a maintenance dose of 10 mg/d over the next 6 months. Benazepril (10 mg/d) or valsartan (80 mg/d, Novartis Pharma, Beijing, China) was administered to other patients.

Aspirin (100 mg/d, Bayer, Germany) or dipyridamole (100 mg/d, Yunpeng Pharmaceutical, Shanxi, China) was provided to patients with a high risk of clotting.

Patients visited the clinic monthly and were followed up for at least 8–12 weeks after the initiation of treatment.

### Blood sampling

Fasting venous blood was collected from all study subjects. For patients with IgAN, blood samples were obtained at the time of kidney biopsy and after 8–12 weeks of treatment. Blood samples from HC were obtained at the same time. Peripheral blood mononuclear cells (PBMCs) were isolated with Ficoll-Paque Plus (Amersham Biosciences, Little Chalfont, UK) from one blood sample collected at each time point; the remaining blood samples were used to prepare serum.

### Flow cytometry analysis

For flow cytometry analysis, PBMCs (10^6^/tube) were stimulated with 50 ng/ml phorbol 12- myristate 13-acetate (PMA) and 1.0 mg/ml of ionomycin (Sigma, St. Louis, MO, USA) in 10% fetal bovine serum (FBS)-RPMI 1640 medium at 37 °C in a humidified incubator at 5% carbon dioxide for 2 h. Cells were cultured in the presence of 0.5 mg/mL brefeldin A (BFA, Sigma) for 4 h, harvested, stained with APC-H7-anti-CD4, BV510-anti-CD3, FITC-anti-CD154, FITC-anti-CD138, PE-Cy7-anti-CD19, PE-Cy7-anti-CD28, BV421-anti-PD1, PE-anti-ICOS and APC-anti-IL21, (BD Biosciences, San Diego, CA), washed with buffer (1 ml per tube; BD Biosciences, San Diego, CA), and centrifuged (250 × g), according to the manufacturer’s, instructions. Controls were isotype-matched antibodies. Cells were washed with PBS and subjected to flow cytometry analysis using a FACSAria II. At least 50,000 events per sample were analyzed using FlowJo software (v5.7.2)^15^.

### ELISA for serum IL-21

Serum IL-21 concentrations were measured using human IL-21 ELISA kits (Biolegend, San Diego, CA, USA), according to the manufacturer’s instructions.

### Cytometric Bead Array (CBA) analysis of serum cytokines

Serum IFN-γ, TNF-α, IL-2, IL-4, IL-10, IL-6, and IL-17A concentrations were determined by CBA^16^, according to the manufacturer’s instructions (BD Biosciences), with minor modifications^17^. Serum cytokine concentrations were quantified on a FACSAria II using the CellQuest Pro and CBA software (Becton Dickinson).

### Statistical analysis

Statistical analyses were performed using GraphPad Prism version 5.01 software. Data are reported as median and range and were analyzed using the Kruskal-Wallis H non-parametric test. The relationship between variables was analyzed using Spearman’s rank correlation test. All tests were two-sided and a P-value of <0.05 was considered significant.

## Results

### Characteristics of the study subjects

This study included 37 patients with IgAN and 23 age- and gender-matched HCs. Demographic and clinical characteristics of the study subjects are shown in Table 1. There were no significant differences in age, gender, leukocyte and lymphocyte counts, serum uric acid, triglyceride, cholesterol, or albumin levels, or microscopic hematuria between patients with IgAN and HCs. However, 24 h urinary protein concentration was higher and eGFR was lower in patients with IgAN, suggesting that patients with IgAN had impaired kidney function.

**Table 1:** Demographic and clinical characteristics of study subjects.

	IgAN (n = 37)	Healthy Controls (n = 23)
Age, year	34 (16–69)	34 (17–69)
Female/Male	22/15	14/9
Serum IgA, g/L	2.88 (1.11–5.98)	2.43 (1.10–4.70)
Urinary proteins, g/24 h	1.67 (0.42–12.10)*	0.05 (0–0.11)
Serum uric acid, μmol/L	396 (143–527)	354.0 (127–460)
Triglycerides, mmol/L	1.46 (0.70–5.53)	1.22(0.50–2.50)
Cholesterol, mmol/L	5.51 (2.80–10.75)	4.67(2.80–6.55)
eGFR, mL/min/1.73 m^2^	77.98 (28.1–137.2)*	101.85 (90.00–126.00)
serum albumin, g/L	33.2 (11.1–47)	42.50 (38.6–50.1)
WBC, 10^9^/L	7.69 (3.57–10.42)	7.03 (4.42–10.21)
Lymphocytes, 10^9^/L	1.21 (0.39–2.68)	1.04 (0.41–1.82)

Data shown are median and range, except when specified. *P < 0.05 vs. the HC.

### Pre-treatment frequency of various subsets of circulating PD1^hi^CXCR5^−^ T cells and CD138^+^ CD19^+^ B cells was higher in patients with IgAN compared to HC*s*

**Figure 1:**
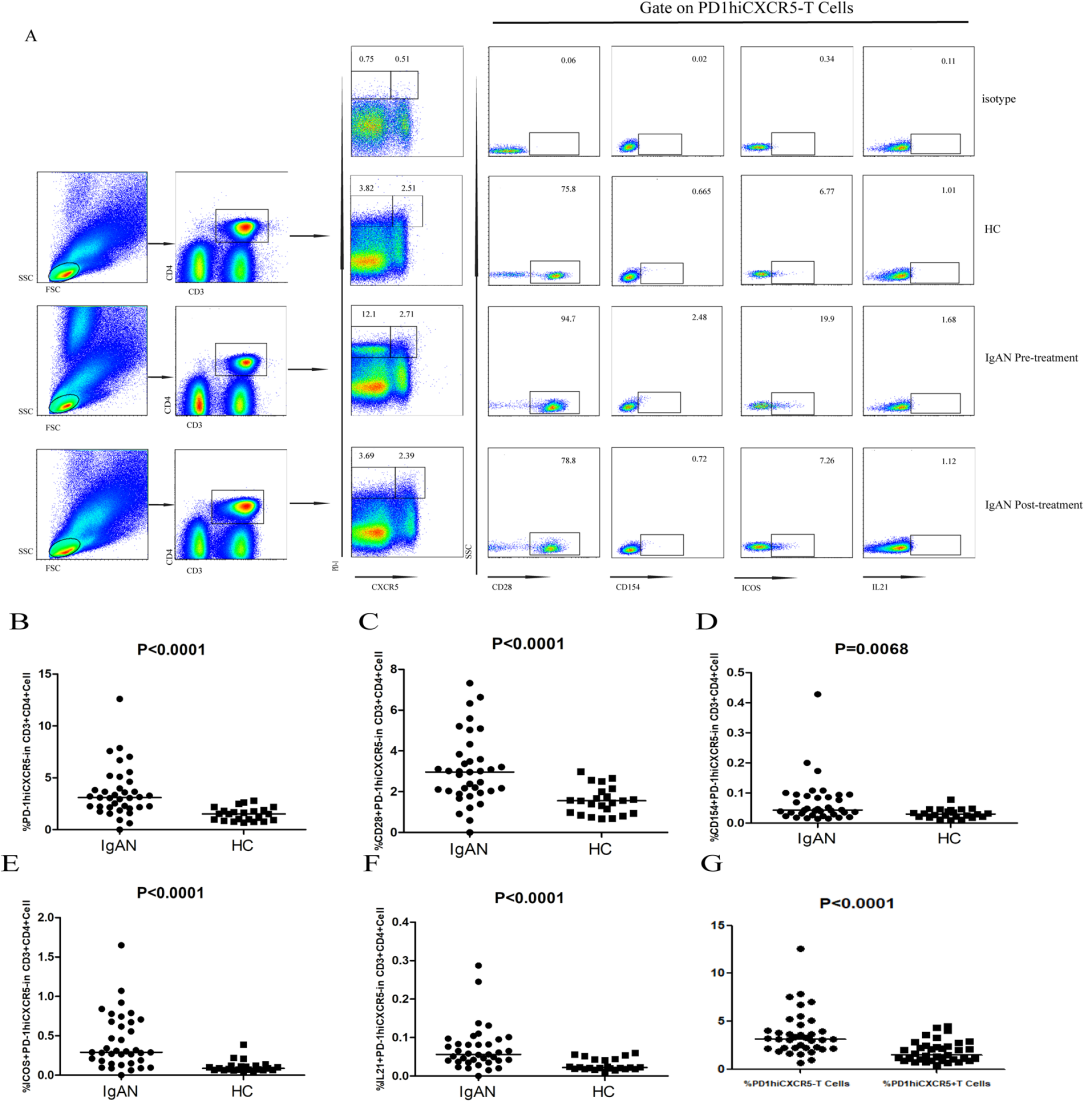
Flow cytometry analysis of the frequency of various subsets of PD1^hi^CXCR5^−^ T cells. PBMCs from patients with IgAN and HCs were stained with anti-CD4, anti-CD3, anti-CXCR5, anti-PD-1, anti-CD28, anti-CD154, anti-ICOS, and anti-IL-21. Cells were gated on living lymphocytes and CD3^+^CD4^+^ T cells. The frequency of PD-1^hi^CXCR5^−^, PD-1^hi^CXCR5^−^ CD28^+^, PD-1^hi^CXCR5^−^CD154^+^, PD-1^hi^CXCR5^−^ICOS^+^, and PD-1^hi^CXCR5^−^IL21^+^ T cells was analyzed by flow cytometry. (**A**) Flow cytometry analysis. (**B**–**G**) Quantitative analyses: data are representative dot plots or are expressed as the mean % of different subsets of Tph and Tfh cells in the total CD3^+^CD4^+^ T cells from individual subjects from three separate experiments. Horizontal lines represent the medians of all subjects.

**Figure 2:**
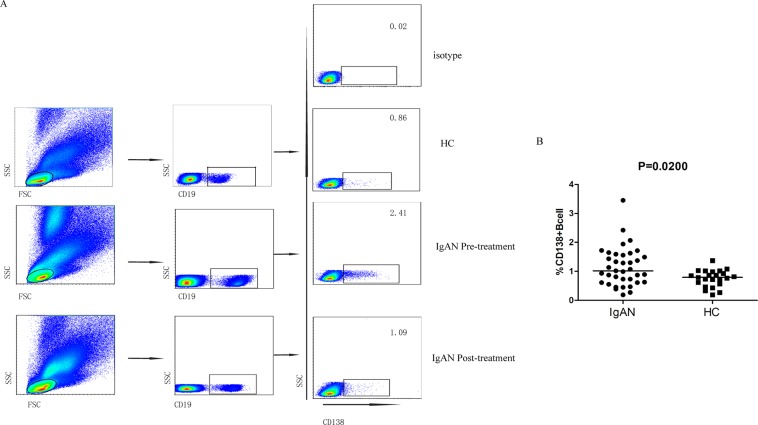
Flow cytometry analysis of plasma cells. PBMCs from patients with IgAN and HCs were stained with anti-CD19, anti-CD138 antibody. Cells were gated on living lymphocytes and CD19^+^ B cells. (**A**) Flow cytometry analysis. (**B**) Quantitative analyses showing CD138^+^CD19^+^ B cells in individual subjects. Horizontal lines represent the medians of all subjects.

There was no significant difference in the percentage of circulating CD3^+^CD4^+^ T cells between patients with IgAN and HCs. However, analysis of the frequency of circulating PD1^hi^CXCR5^−^ T cells indicated that the percentage of circulating PD1^hi^CXCR5^−^ (p < 0.0001), CD28^+^PD-1^hi^CXCR5^−^ (p < 0.0001), CD154^+^PD-1^hi^CXCR5^−^ (p = 0.0068), ICOS^+^PD-1^hi^CXCR5^−^ (p < 0.0001), and IL21^+^PD-1^hi^CXCR5^−^ (p < 0.0001) T cells was significantly higher in patients with IgAN compared to HCs (Fig. 1B–F), and that the percentage of circulating PD1^hi^CXCR5^−^ T cells was significantly higher than the percentage of circulating PD1^hi^CXCR5^+^ T cells in patients with IgAN (p < 0.0001; Fig. 1G). Furthermore, the percentage of circulating CD138^+^CD19^+^ B cells was significantly higher in patients with IgAN compared to HCs (p = 0.0200; Fig. 2B).

### Associations between different subsets of PD1^hi^CXCR5^−^ T cells, CD138^+^ CD19^+^ B cells, and clinical parameters in patients with IgAN

**Figure 3:**
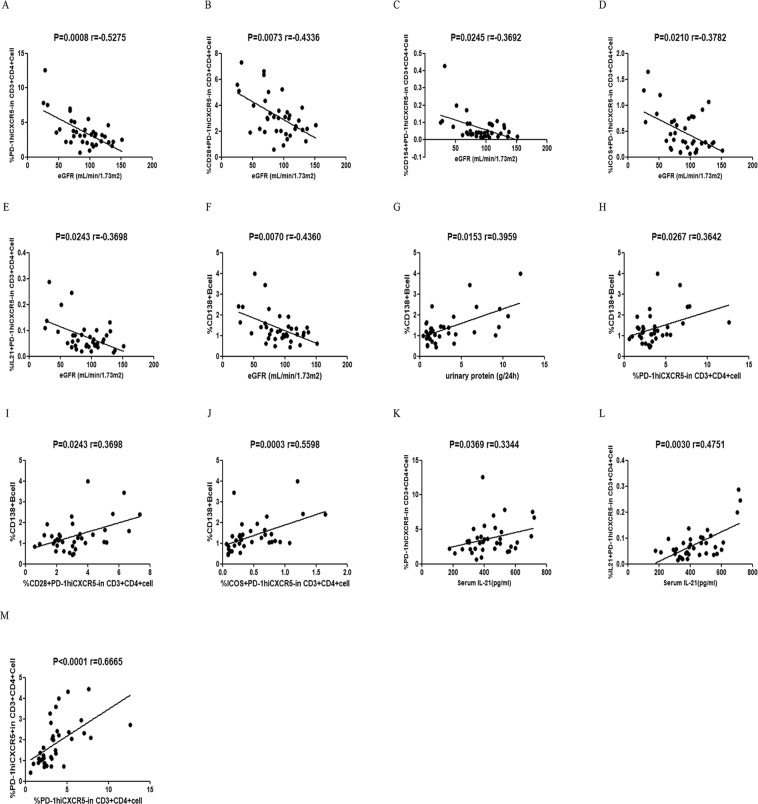
Correlations between the frequency of various subsets of circulating PD1^hi^CXCR5^−^ T cells, circulating CD138^+^CD19^+^ plasma cells, and clinical parameters in patients with IgAN. (**A**–**F**) eGFR was negatively correlated with the percentage of the different subsets of circulating PD1^hi^CXCR5^−^ T cells and CD138^+^ B cells; (**G**–**J**) The percentage of circulating CD138^+^ B cells was positively correlated with 24-h urinary protein concentration and the percentage of circulating PD-1^hi^CXCR5^−^, PD-1^hi^CXCR5^−^CD28^+^, PD-1^hi^CXCR5^−^ICOS^+^ T cells; (**K**,**L**) The percentage of PD-1^hi^CXCR5^−^ and PD-1^hi^CXCR5^−^IL-21^+^ T cells was positively correlated with serum IL-21 concentration; (**M**) The percentage of PD-1^hi^CXCR5^−^ T cells was positively correlated with the percentage of PD-1^hi^CXCR5^+^ T cells.

The percentage of circulating PD1^hi^CXCR5^−^ (r = −0.5275, p = 0.0008), CD28^+^PD-1^hi^CXCR5^−^ (r = −0.4336, p = 0.0073), CD154^+^PD-1^hi^CXCR5^−^ (r = −0.3692, p = 0.0245), ICOS^+^PD-1^hi^CXCR5^−^ (r = −0.3782, p = 0.0210), and IL21^+^PD-1^hi^CXCR5^−^ (r = −0.3689, p = 0.0243) T cells and CD138^+^ CD19^+^ B cells (r = −0.4360, p = 0.0070) were negatively correlated with eGFR (Fig. 3A–F). The percentage of circulating CD138^+^ CD19^+^ B cells was positively correlated with the 24 h urinary protein concentration (r = 0.3959, p = 0.0153) (Fig. 3G). The percentage of circulating PD1^hi^CXCR5^−^ (r = 0.3642, p = 0.0267), CD28^+^PD-1^hi^CXCR5^−^ (r = 0.3689, p = 0.0243), and ICOS^+^PD-1^hi^CXCR5^−^ (r = 0.5598, p = 0.0003) T cells were positively correlated with the percentage of CD138^+^ CD19^+^ B cells (Fig. 3H–J). The percentage of circulating PD1^hi^CXCR5^−^ (r = 0.3344, p = 0.0369) and IL21^+^PD-1^hi^CXCR5^−^ (r=0.4751, p = 0.0030) T cells were positively correlated with serum IL-21 concentrations (Fig. 3K,L). The percentage of circulating PD1^hi^CXCR5^−^ T cells was positively correlated with the percentage of circulating PD1^hi^CXCR5^+^T cells (r = 0.6655, p < 0.0001; Fig. 3M).

### Pre-treatment serum IL-4, IL-17A, IL-10, IFN-γ, and IL-21 concentrations were enhanced in patients with IgAN compared to HCs

**Figure 4:**
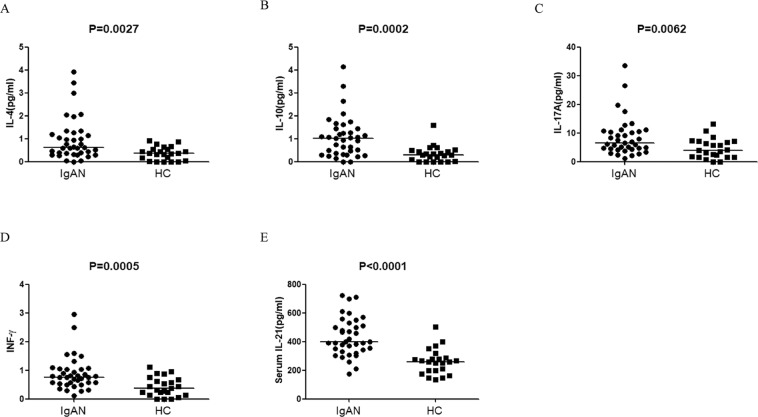
Serum cytokine concentrations. (**A**–**E**) Serum Il-4, IL-10, IL-17A, IFN-γ and IL-21 concentrations in patients with IgAN and HCs were detected by CBA and ELISA. Data are expressed as the mean values of individual samples from three separate experiments. Horizontal lines represent the median values.

Pretreatment serum IL-4 (p = 0.0027), IL-10 (p = 0.0002), IL-17A (p = 0.0062), IFN-γ (p = 0.0005), and IL-21 (p < 0.0001) concentrations were significantly higher in patients with IgAN compared to HCs (Fig. 4A–E)

### Frequency of different subsets of PD1^hi^CXCR5^−^ T cells and CD138^+^ CD19^+^ B cells, serum cytokine concentrations, and clinical parameters in patients with IgAN following treatment

**Figure 5:**
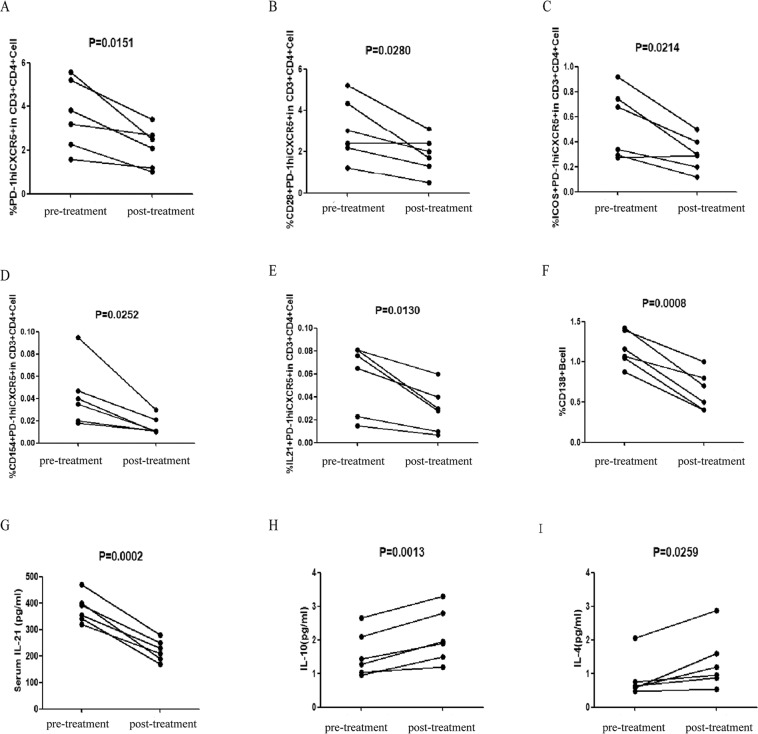
Altered frequency of TPH cells, B cells and levels of serum cytokines in IgAN patients after treatment. The percentages of different subsets of TPH cells and the levels of serum cytokines were compared in IgAN patients before and after the treatment. Data are expressed as the mean % or concentrations of individual subjects from two separate experiments. The numbers of circulating PD-1^+^, CD28^+^, CD154^+^, ICOS^+^, IL-21^+^ TFH cells, CD138^+^CD19^+^ B cells and the level of serum IL-21 of individual patients in the pre- and post-treatment stages, (**A**–**G**) respectively. The levels of serum IL-4 and IL-10 of individual patients in the pre- and post-treatment stages, (**H**–**I**) respectively.

Six patients were followed-up for 8–12 weeks after the initiation of treatment. Among these patients, treatment resulted in a significant reduction in the percentage of circulating PD-1^hi^CXCR5^−^ (p = 0.151), CD28^+^PD-1^hi^CXCR5^−^ (p = 0.0280), CD154^+^PD-1^hi^CXCR5^−^ (p = 0.0252), ICOS^+^PD-1^hi^CXCR5^−^ (p = 0.0214), and IL21^+^PD-1^hi^CXCR5^−^ (p = 0.0130) T cells, CD138^+^ CD19^+^ B cells (p = 0.0008), and serum IL-21 concentration (p = 0.0002) (Fig. 5A–G). In addition, there was a significant decrease in 24-h urinary protein concentration, but eGFR was significantly elevated (p < 0.05; Table 2). Treatment resulted in a significant increase in serum IL-10 (p = 0.0259) and IL-4 concentrations (p = 0.0013, Fig. 5H,I). There were no significant differences in the concentrators of other serum cytokines before and after treatment (data not shown).

**Table 2:** The effect of treatment on clinical parameters in patients with IgAN.

	Before treatment	After treatment
Age, years	40(26–58)	40 (26–58)
Female/Male	4/2	4/2
Serum IgA, g/L	3.26 (1.79–4.88)	3.09 (2.18–3.75)
Urinary protein, g/24 h	5.19 (0.98–9.50)	2.61 (0.47–5.54)*
Serum uric acid, μmol/L	381 (326–451)	369.83 (217–431)
Triglycerides, mmol/L	1.85 (0.90–3.12)	1.79 (1.12–2.98)
Cholesterol, mmol/L	6.92 (2.80–9.95)	5.85 (2.67–8.33)
eGFR, mL/min/1.73 m^2^	66.89 (32.1–119)	86.83 (41.3–115)*
serum albumin, g/L	24.8 (12.1–31)	31.72 (27.3–40.6)
WBC, 10^9^/L	6.44 (4.96–8.12)	6.79 (5.37–8.44)
Lymphocytes, 10^9^/L	1.56 (1.21–2.58)	1.49 (0.37–2.34)

Data are present as median (range). *P < 0.05 vs. the values before treatment.

## Discussion

This study described the percentage of various subsets of circulating PD-1^hi^CXCR5^−^ T cells and CD138^+^ B cells in adults with IgAN and HCs. Findings showed that the percentage of different subsets of circulating PD-1^hi^CXCR5^−^ T cells and CD138^+^ B cells were significantly increased in patients with IgAN compared to HCs. To understand the roles of circulating PD-1^hi^CXCR5^−^ T cells and CD138^+^ B cells in the progression of IgAN, we investigated potential associations between the percentages of different subsets of circulating PD1^hi^CXCR5^−^ T cells, CD138^+^ CD19^+^B cells, and clinical parameters in patients with IgAN. Results showed that the percentage of circulating PD1^hi^CXCR5^−^, CD28^+^PD-1^hi^CXCR5^−^, CD154^+^PD-1^hi^CXCR5^−^, ICOS^+^PD-1^hi^CXCR5^−^, and IL21^+^PD-1^hi^CXCR5^−^ T cells and CD138^+^ CD19^+^ B cells were negatively correlated with eGFR. The percentage of circulating CD138^+^ CD19^+^ B cells was positively correlated with the 24 h urinary protein concentration. IgAN treatment significantly reduced the frequency of circulating PD-1^hi^CXCR5^−^ T cells and CD138^+^ B cells. These findings support the hypothesis that different subsets of circulating PD-1^hi^CXCR5^−^ T cells and CD138^+^ B cells contribute to the progression and pathogenesis of IgAN and show that treatment dramatically improved the clinical parameters of IgAN disease.

Evidence suggests that abnormal T- and/or B-cell expression has an important role in the pathophysiology of immune diseases. A previous report revealed that PD-1^hi^CXCR5^−^ T cells secrete IL-21 and promote plasma cell differentiation, similar to PD-1^hi^CXCR5^+^ Tfh cells^9^. Other studies have identified a role for T- and B-cell dysregulation in the pathophysiology of autoimmune diseases^18–20^, and more specifically, kidney damage in minimal change nephropathy, systemic lupus erythematosus, and membranous nephropathy^21^.

Some research shows that T cells with a high expression of programmed cell death protein-1 (PD-1) have impaired cytotoxicity^22–25^, while other reports suggest that PD-1^+^T cells induce high levels of autoantibody production by activating antigen-specific autoreactive B cells and promoting the survival of long-lived plasma cells^26^. In the present study, the percentage of different subsets of circulating PD-1^hi^CXCR5^−^ T cells and CD138^+^ B cells was significantly higher in patients with IgAN compared to HCs, and the percentage of circulating CD138^+^ B cells was positively correlated with the percentage of circulating PD1^hi^CXCR5^−^, CD28^+^PD-1^hi^CXCR5^−^, and ICOS^+^PD-1^hi^CXCR5^−^ T cells. These data suggest that PD-1^hi^CXCR5^−^ T cells support B-cell responses and antibody production by mechanisms that are dependent on costimulation through CD28 or includible costimulator (ICOS). Accordingly, previous studies demonstrated that CD28 is a costimulatory receptor that binds CD80 and CD86 and plays an important role in T cell-B cell interactions^27^, and ICOS costimulation in necessary for T cell proliferation and is involved in humoral immune responses (B cell germinal center formation)^28^.

IL-21 production is mostly restricted to Tfh cells, and is important for Tfh cell differentiation^29^. However, some evidence shows that PD-1^hi^CXCR5^−^ T (Tph) cells also express and release IL-21^9^ to recruit B cells and Tfh cells and promote the production of autoantibodies^30,31^. One report showed that plasma cell differentiation can be induced *in vitro* by Tph cells through IL-21 secretion and surface molecule interaction^9^. In this study, we found a positive correlation between the percentage of circulating PD-1^hi^CXCR5^−^ and IL21^+^PD-1^hi^CXCR5^−^ T cells and serum IL-21 concentrations. Most importantly, serum IL21 concentrations were significantly higher in patients with IgAN than HCs.

Corticosteroids are widely used as immunotherapy as they inhibit the T/B response and the production of cytokines. In this study, corticosteroid treatment of patients with IgAN significantly reduced the percentage of circulating PD-1^hi^CXCR5^−^ T cells and CD138^+^ B cells, as well as serum IL-21 concentration; these findings align with those of a previous study^11^. Th1 cells selectively produce IFN-γ and IL-2 and increase cell-mediated immunity. Th2 cells selectively produce IL-4 and IL-10 and are responsible for antibody production. IL-17A is a characteristic cytokine of Th17 cells with a key role in the pathogenesis of chronic inflammatory diseases and autoimmune responses^32^. In this study, serum IL-4, IL-10, IL-17A, and IFN-γ concentrations were significantly higher in patients with IgAN compared to HCs. We speculate that Th1 responses may be involved in the mechanism of IgAN and induce anti-inflammatory Th2 cells, which feedback to down regulate proinflammatory responses during the pathogenesis of IgA. This hypothesis is consistent with previous reports^33^. After treatment, serum IL-10 and IL-4 concentrations were significantly increased in patients with IgAN, while the concentrations of other cytokines were unchanged. Taken together, these data suggest that pro-inflammatory Th1 and Th17 responses may be involved in the pathogenesis of IgAN and the anti-inflammatory Th2 response may predominate after corticosteroid treatment.

We recognize that our study has limitations, including a small sample size and the lack of functional investigations exploring different types of PD-1^hi^CXCR5^−^ T cells and B cells. Thus, further studies in a larger population are warranted.

In conclusion, our study revealed that patients with IgAN have higher levels of circulating PD-1^hi^CXCR5^−^ T cells and B cells than HCs, and the percentage of these cells is correlated with disease severity. These findings offer new insights into understanding the pathogenesis of IgAN. Furthermore, the high expression of PD-1 on Tph cells in patients with IgAN represents a potential strategy for therapeutic targeting.
